# Economic impact of the Elecsys anti-Müllerian hormone Plus immunoassay for anti-Müllerian hormone testing as part of polycystic ovary syndrome assessment in the United Kingdom

**DOI:** 10.1371/journal.pone.0326162

**Published:** 2025-06-17

**Authors:** Osvaldo Ulises Garay, Anna-Maria Olziersky, Joop Laven, Rebecca Mawson, Terhi Piltonen, Stephen Franks, Johanna Sillman

**Affiliations:** 1 Roche Diagnostics International Ltd, Rotkreuz, Switzerland; 2 Division of Reproductive Endocrinology and Infertility, Erasmus University Medical Center, Division of Reproductive Medicine Department of Obstetrics & Gynaecology, Rotterdam, Netherlands; 3 Division of Population Health, The University of Sheffield, Sheffield, United Kingdom; 4 Department of Obstetrics and Gynecology, Research Unit of Clinical Medicine and Medical Research Center, Oulu University Hospital and University of Oulu, Oulu, Finland; 5 Institute of Reproductive and Developmental Biology, Imperial College London, London, United Kingdom; Shiraz University of Medical Sciences, IRANISLAMIC REPUBLIC OF

## Abstract

Polycystic ovary syndrome (PCOS) is the most common endocrine disorder among women. Current international PCOS assessment and management guidelines recommend anti-Müllerian hormone (AMH) as an alternative to transvaginal ultrasound for assessing polycystic ovarian morphology, which is one of three criteria for diagnosing PCOS. This study assessed the economic impact of using the Elecsys® AMH Plus immunoassay (Roche Diagnostics International Ltd, Rotkreuz, Switzerland) for AMH testing in the United Kingdom health system to assess women with signs and symptoms of PCOS. A decision tree model estimated the costs and health outcomes of using the Elecsys AMH Plus immunoassay to determine polycystic ovarian morphology as part of PCOS assessment in a simulated cohort of women aged 25–45 years who were exposed to different diagnosis pathways. The comparator scenario was the standard of care, where transvaginal ultrasound was used for assessment. Base-case results indicated that the Elecsys AMH Plus immunoassay could lead to cost savings of £284,029 per year on the total cost of PCOS diagnosis (1.4% reduction vs. transvaginal ultrasound), in addition to savings on managing secondary comorbidities, such as type 2 diabetes and stroke care. Cost savings with the Elecsys AMH Plus immunoassay were observed in all scenarios versus using transvaginal ultrasound, including scenarios with various referral rates to specialists and dropout rates from the diagnosis pathway, and low adherence to lifestyle recommendations. With the known current delays in the United Kingdom for diagnosis of PCOS, implementing the Elecsys AMH Plus immunoassay for AMH testing may not only provide cost benefits, but also reduce waiting times for diagnosis and treatment, improving patient health outcomes.

## Introduction

Polycystic ovary syndrome (PCOS) is a heterogeneous endocrine and metabolic disorder, characterized by hyperandrogenism (HA) and ovarian dysfunction [1,2]. PCOS is one of the most common endocrine disorders among premenopausal women, with a reported prevalence ranging between 6% and 20%, depending on the diagnostic criteria used [2–4]. Although PCOS affects many women, the condition is under-represented in clinical trials and in economic evaluations of diagnostic pathways [5]. Globally, the burden of disease of PCOS has increased, with the incidence of PCOS and the associated disability-adjusted life years having increased by 54% and 91%, respectively, from 1990 to 2019 [4]. In particular, the global burden of PCOS varies by age group, race, and socio-demographic index quintiles, as well as across specific countries and territories, reflecting health inequality and the obstacles that exist across populations to access health resources [4].

In addition to the common symptoms of PCOS, such as irregular menstruation, hirsutism, and acne [6], further complications and comorbidities can occur that contribute to the long-term burden of PCOS [7–10]. For instance, compared with individuals without PCOS, individuals with PCOS are at increased risk of type 2 diabetes (T2D) and metabolic disturbances such as dyslipidemia and hypertension; adverse pregnancy outcomes; psychological conditions, such as depression and anxiety; and endometrial cancer [7,8,11,12]. In addition, there is a heavy economic burden for patients with PCOS and the associated health systems, with additional costs for pregnancy-related and long-term comorbidities [13].

The diagnosis of PCOS is typically based on the Rotterdam criteria, where any two of the following three characteristics must be met: oligo-/anovulation (OA), HA, and polycystic ovarian morphology (PCOM) based on antral follicle count (AFC) via transvaginal ultrasound (TVUS) [14,15]. This is consistent with the National Institute for Health and Care Excellence United Kingdom (UK) guidelines [16]. However, these criteria each pose their own challenges, which can make the accurate diagnosis of PCOS difficult. For instance, for initial diagnosis, OA and HA are often determined through subjective measures, such as tracking the menstrual cycle or clinical evaluations of hair growth, respectively; as such, PCOS can often go unrecognized since many women are treating these initial symptoms at home [17]. The PCOM criterion can be especially difficult to implement due to limitations associated with using TVUS; for example, limited accessibility to TVUS in the primary care setting, contributing to delayed diagnosis and under-diagnosis of PCOS [18,19], and limited assessment of PCOM in certain demographics due to occurrence of polycystic ovaries in adolescents without PCOS, as well as cultural and religious factors [20]. When TVUS is available, it requires an expert sonographer for reliable assessment of AFC, and inter-observer agreement has been found to be moderate to poor [17,19,21]. Moreover, guidelines may be interpreted differently, leading to differences in diagnosis pathways and variations in the care provided between different countries [18]. PCOS is an under-recognized and under-diagnosed condition [22], highlighting the need for advancements in diagnostic solutions.

Considering the challenges associated with diagnosing PCOS, and in particular the assessment of PCOM, the 2023 International Evidence-based Guidelines for the Assessment and Management of PCOS now recommends measuring serum levels of anti-Müllerian hormone (AMH) as an alternative to TVUS for defining PCOM [23]. AMH is a glycoprotein expressed by the granulosa cells of the pre-antral and small antral follicles in the ovaries [24]. Serum AMH levels have been shown to correlate with the number of antral follicles and are higher in women with PCOS, who are also fulfilling the PCOM criterion, compared with those without PCOS [19,24,25]. The APHRODITE (AMH Protein in Humans for polycystic ovaRian mOrphology DIagnostic TEsting) study showed that using the Elecsys® AMH Plus immunoassay (Roche Diagnostics International Ltd, Rotkreuz, Switzerland) cut-off of 3.2 ng/mL provided a high sensitivity and specificity for identifying PCOM [19]. This was further validated in a large population-based study where the cut-off of 3.2 ng/mL resulted in a prevalence of PCOS in line with previous general population-based studies [25]. As such, measurement of serum AMH levels is a possible substitute for AFC measured by TVUS in the identification of PCOM as part of the PCOS assessment in adult women, who show OA or HA [23]. Although the 2023 International Evidence-based Guidelines state that testing for AMH could be used as a replacement for TVUS, its economic impact in the UK health system is uncertain.

In this study, the objective was to assess the economic impact of using the Elecsys AMH Plus immunoassay for AMH testing in the UK to identify PCOM as part of PCOS assessment in women with signs and symptoms, using a health economics model. The model utilized a decision tree structure, which assumed the 2023 International Evidence-based Guidelines were followed, and was conducted from the perspective of the UK National Health Service (NHS).

## Methods

### Study overview

A decision tree model was developed in Microsoft Excel to estimate the costs and health outcomes of using the Elecsys AMH Plus immunoassay to diagnose PCOM, across a lifetime horizon, in a simulated cohort of women aged 25–45 years who were exposed to different diagnosis pathways in the UK. The model was created in collaboration with clinical experts to represent the typical patient pathway for those with signs and symptoms of PCOS, if recommendations of the 2023 International Evidence-based Guidelines were followed [23]. The study perspective was the UK NHS. This article follows the Consolidated Health Economic Evaluation Reporting Standards guidelines on reporting economic evaluations [26].

### Model structure

**Fig 1:**
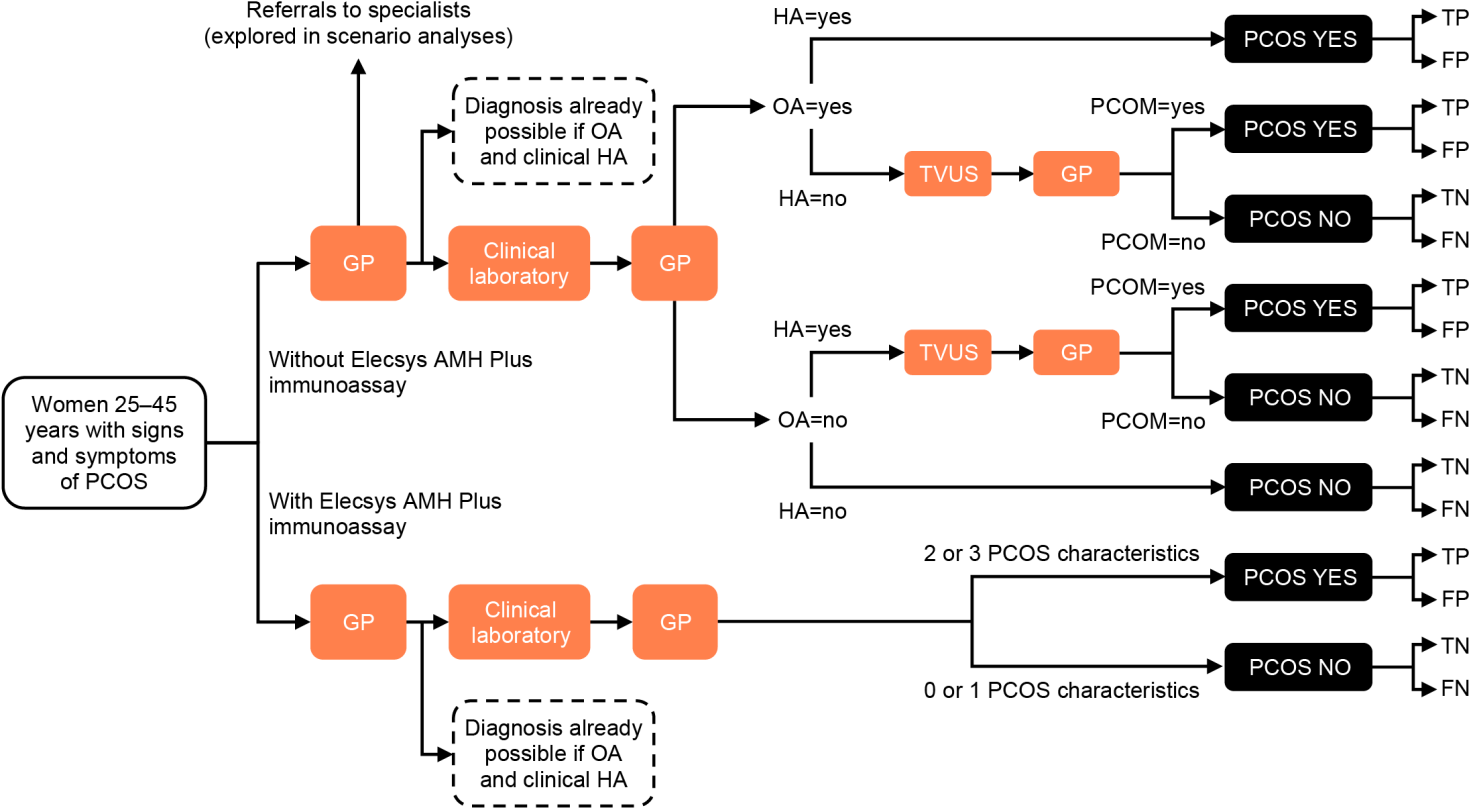
Model representing the typical patient pathway for patients with signs and symptoms of PCOS. AMH, anti-Müllerian hormone; FN, false negative; FP, false positive; GP, general practitioner; HA, hyperandrogenism; OA, oligo-/anovulation; PCOM, polycystic ovarian morphology; PCOS, polycystic ovary syndrome; TN, true negative; TP, true positive; TVUS, transvaginal ultrasound.

The model structure (Fig 1) and main assumptions were discussed in a series of face-to-face and online feedback sessions with clinical experts. The comparator scenario was the standard of care, without the Elecsys AMH Plus immunoassay, where TVUS for AFC was utilized to diagnose PCOM as part of the PCOS diagnostic process, in accordance with the 2023 International Evidence-based Guidelines [23].

Patients entered the model with signs and symptoms of PCOS, with their first point of contact for diagnosis being a general practitioner (GP). In the base-case scenario, it was assumed that GPs did not refer patients to specialists until PCOS was diagnosed, but different referral scenarios were explored. At the first consultation, the GP evaluated OA, clinical HA, and requested a set of laboratory tests to assess biochemical HA and exclude other conditions. If both OA and HA were confirmed or discarded, PCOS was diagnosed or discarded, respectively, and a TVUS for AFC was not required. However, if either one of OA or HA were confirmed, then a PCOM diagnosis was explored. It was assumed in this case that a TVUS was ordered for the patient, who later returned to the GP for confirmation of PCOS diagnosis depending on the diagnosis of PCOM. In the intervention scenario, the Elecsys AMH Plus immunoassay was requested alongside the other laboratory tests and, at the following consultation, a diagnosis was made based on the results.

In our model, individuals with PCOS were assumed to have an increased risk of T2D and stroke compared with individuals without PCOS. If a diagnosis of PCOS was made, the patient received a lifestyle intervention. If the diagnosis was a true positive, the individual benefited from a risk reduction in T2D, stroke, and the associated event costs based on an expected effectiveness of the lifestyle intervention received. If the diagnosis was a false positive, we assumed no consequences from the lifestyle interventions more than the costs associated. If the diagnosis was a true negative, no consequences after the diagnosis were considered, but if it was a false negative, the individual was assumed to be exposed to a higher risk of T2D, stroke, and the associated event costs.

### Model parameters

The model was populated with data from diverse sources, including published literature, unit costs registries, and expert opinions. Table 1 shows the parameters used to perform all analyses, which included the base-case values to obtain results for the deterministic base-case, the minimum and maximum values used for sensitivity analyses, and the respective data sources.

**Table 1:** Model parameters used to perform all analyses, including the base-case values to obtain results for the deterministic base-case, the minimum and maximum values used for sensitivity analyses, and the respective data sources.

Parameters	Base (min–max)	Sources
Women aged 25–45 years, n	9,145,746	Office for National Statistics UK [30]
Incidence of signs and symptoms, n per 100,000	142 (106.5–177.5)	Calibrated using Liu (2021) [27], Gabrielli (2012) [28] and Lizneva (2016) [29]
Annual incident cases, n per 100,000	78.6 (61.2–95.9)	Liu (2021) [27]
**Distribution of population, %**		
OA = yes	54.7	Gabrielli (2012) [28]
HA = yes, if OA = yes	46.0	Calibrated using Lizneva (2016) [29] and Gabrielli (2012) [28]
PCOM = yes, if HA = yes & OA = yes	44.0	Calibrated using Lizneva (2016) [29] and Gabrielli (2012) [28]
PCOM = yes, if HA = no & OA = yes	37.0	Calibrated using Lizneva (2016) [29] and Gabrielli (2012) [28]
HA = yes, if OA = no	78.0	Calibrated using Lizneva (2016) [29] and Gabrielli (2012) [28]
PCOM = yes, if HA = yes & OA = no	54.0	Calibrated using Lizneva (2016) [29] and Gabrielli (2012) [28]
Clinical.HA = no and Biochemical.HA = yes	9.0 (7.2–10.8)	HARMONIA (internal analysis) [31]
**Phenotype distribution, %**		
A: HA, OA, and PCOM	19.0	Lizneva (2016) [29]
B: HA, OA	25.0	Lizneva (2016) [29]
C: HA, PCOM	34.0	Lizneva (2016) [29]
D: OA, PCOM	19.0	Lizneva (2016) [29]
**Sensitivity and specificity for PCOM, %**		
Sensitivity of Elecsys AMH Plus immunoassay	88.6 (85.3–91.3)	APHRODITE [19]
Specificity of Elecsys AMH Plus immunoassay	84.6 (81.1–87.7)	APHRODITE [19]
Sensitivity of TVUS	85.1 (82.3–100.0)	Meta-analysis, assuming 100% accuracy in the upper range limit (see Methods)
Specificity of TVUS	92.4 (88.7–100.0)	Meta-analysis, assuming 100% accuracy in the upper range limit (see Methods)
**Risk, %**		
Excess risk T2D	3.9 (2.9–4.9)	Riestenberg (2022) [13]
Excess risk stroke	4.3 (3.2–5.4)	Riestenberg (2022) [13]
Relative risk reduction: long-term efficacy of lifestyle interventions	36.5 (0.0–61.4)	Based on Costa (2012) [32] & Lindström (2012) [33]
**Unit cost, £**		
Elecsys AMH Plus immunoassay	40.0 (28.0–60.0)	Roche Diagnostics UK. Base-case is a list price. Ranges were calculated as +/- 15 GBP
TVUS scan by diagnostic imaging service	61.0 (49.0–73.0)	National Cost Collection for the NHS, 2021/2022 [34]
TVUS scan by gynecology service	236.0 (188.0–283.0)	National Cost Collection for the NHS, 2021/2022 [34]
Outpatient appointment OBGYN	181.0 (149.0–186.0)	National Cost Collection for the NHS, 2021/2022 [34]
Outpatient appointment GP	22.0 (18.0–41.0)	Unit Costs of Health and Social Care 2022 [35]
Dermatology/endocrinology outpatient appointment	165.0 (136.0–169.0)	National Cost Collection for the NHS, 2021/2022 [34]
Cost of lifestyle interventions	363.0 (272.0–453.0)	Estimated using unpublished data from the Sheffield City Council weight loss program
Discounted lifetime cost of T2D	34,518 (25,889–43,148)	Wang (2022) [37]
Discounted lifetime costs of stroke	46,039 (41,432–52,726)	Xiang-Ming (2018) [38]

AMH, anti-Müllerian hormone; GBP, Great British pound; GP, general practitioner; HA, hyperandrogenism; NHS, National Health Service; OA, oligo-/anovulation; OBGYN, obstetrics and gynecology; PCOM, polycystic ovarian morphology; T2D, type 2 diabetes; TVUS, transvaginal ultrasound; UK, United Kingdom.

The number of patients with signs and symptoms of PCOS was estimated by applying a calculated incidence rate of signs and symptoms to the overall population of women aged 25–45 years in the UK. The age group was defined to match the population of the APHRODITE study [19]. The incidence rate of signs and symptoms was estimated using the annual incidence rate for PCOS, a calibrated distribution of PCOS characteristics, and the distribution of PCOS phenotypes A − D (Table 1) [27–30]. In the absence of data, the incidence rate of signs and symptoms, and the distribution of polycystic ovary syndrome (PCOS) characteristics in women with signs and symptoms, were estimated through a calibration process. The calibration consisted of finding a combination for the distribution of PCOS characteristics and the incidence of signs and symptoms of PCOS so the model predictions matched a group of selected targets. Further explanation on the calibration is provided in the Supporting Information, Supplementary Methods and S1–3 Tables in S1 Appendix. The distribution of woman without clinical presentation of HA, but with a confirmed diagnosis of HA was obtained from an internal analysis using the HARMONIA cohort [31].

Regarding the diagnostic performance of TVUS for PCOS, we meta-analyzed sensitivity and specificity estimates of TVUS for PCOS using all the studies identified in the systematic review of the 2023 International Evidence-based Guidelines [23] used to discuss the performance of TVUS. In addition, we conservatively assumed a perfect diagnostic performance for the upper limit of the parameter range (100% sensitivity, 100% specificity) for the sensitivity analyses. Further information is provided in the Supporting Information, Supplementary Methods, S1 Fig in S1 Appendix and S4–S6 Tables in S1 Appendix. The diagnostic performance of the Elecsys AMH Plus immunoassay was obtained from the APRHODITE study [19].

The excess risk of T2D and stroke were obtained from a model-based study that looked to estimate the economic burden of PCOS in the United States [13]. For the effectiveness of lifestyle interventions in reducing the risk of T2D and stroke, we followed a pragmatic approach assuming an expected risk reduction of 36.5% based on the studies of Costa 2012 [32] and Lindstrom 2012 [33] (varied from 0–61.4% in the parameter range), assuming a 100% adherence rate for the lifestyle recommendations.

The unit cost estimations for TVUS and outpatient appointments with obstetrics and gynecology and dermatology/endocrinology were based on data from the National Cost Collection for the UK NHS, 2021/2022 [34]. For the expected cost of a GP consultation, the Personal Social Services Research Unit, Unit Costs of Health and Social Care 2022 was used [35]. The unit cost of an Elecsys AMH Plus test was informed by Roche Diagnostics UK. The cost of a lifestyle intervention per person was estimated using unpublished data from the Sheffield City Council weight loss program (a 12-week program to reduce weight via the adoption of lifestyle initiatives) [36], with a budget of approximately £190,000 for 524 adults in 2022 (~£362 per completer), considering +/ − 25% variations for variability range. Finally, the discounted lifetime event costs of T2D and stroke were obtained from the studies of Wang 2022 [37], a matched-cohort study with 6,383 individuals in the UK with T2D, and Xiang-Ming 2018 [38], a model-based analysis using registries from 84,184 individuals from England, Wales, and Northern Ireland.

### Sensitivity and scenario analyses

The impact of parameter uncertainty on the results was explored using deterministic and probabilistic sensitivity analyses. The deterministic sensitivity analysis, which allowed the identification of the drivers of the results, was analyzed in the form of tornado plots. The probabilistic sensitivity analysis was a joint assessment of the impact of the uncertainty associated with the parameters and consisted of simulating the result 10,000 times using Monte Carlo simulations, assuming that the variation over the ranges of parameters had been distributed according to specific probability distributions. The latter were selected according to technical recommendations [39], with the distribution parameters estimated from the means and standard deviations using the method of moments.

The following scenario analyses were explored:

- **With TVUS for AFC (if TVUS is requested for all PCOS suspicions) versus with the AMH test (base-case):** this scenario assumed that due to delays in TVUS access, GPs requested both laboratory tests and TVUS at the first consultation, and that there was a second consultation to discuss laboratory results, as well as a third to discuss the TVUS
- **With TVUS for AFC (base-case) versus with the AMH test (if test is required only after HA):** this scenario assumed that the Elecsys AMH Plus immunoassay was required only after the first set of laboratory tests, and only for women requiring a PCOM diagnosis to rule-out or rule-in PCOS
- **With TVUS for AFC (if GPs had a low referral rate to specialists) versus with the AMH test (base-case):** this scenario assumed that 15% of GPs referred patients with suspicions of PCOS (10% to endocrinologists or dermatologists and 5% to gynecologists)
- **With TVUS for AFC (if GPs had a high referral rate to specialists) versus with the AMH test (base-case):** this scenario assumed that 30% of GPs referred patients with suspicions of PCOS (20% to endocrinologists or dermatologists and 10% to gynecologists)
- **With TVUS for AFC (considering a 10% drop out rate before TVUS) versus with the AMH test (base-case, no drop out):** this scenario assumed that 10% of patients drop out of the diagnosis pathway when a TVUS is requested to evaluate PCOM. In the Elecsys AMH Plus immunoassay scenario, the drop out rate was assumed to be 0%
- **With TVUS for AFC (considering a 25% drop out rate before TVUS) versus with the AMH test (base-case, no drop out):** this scenario assumed that 25% of patients drop out of the diagnosis pathway when a TVUS is requested to evaluate PCOM. In the Elecsys AMH Plus immunoassay scenario, the drop out rate was assumed to be 0%
- **With TVUS for AFC (considering a 50% drop out rate before TVUS) versus with the AMH test (base-case, no drop out):** this scenario assumed that 50% of patients drop out of the diagnosis pathway when a TVUS is requested to evaluate PCOM. In the Elecsys AMH Plus immunoassay scenario, the drop out rate was assumed to be 0%
- **Lower adherence rate for the lifestyle recommendations:** this scenario assumed that only 50% of patients adhered to the lifestyle recommendations, reducing the efficacy of the lifestyle interventions by 50%.

An additional scenario analysis was performed to replace the calibration estimates with data from the HARMONIA study [31] to assess the potential impact of the calibration methods on the estimation of PCOS characteristics in women with signs and symptoms of PCOS. Further detail can be found in the Supporting Information, Supplementary Methods and S7–S9 Tables in S1 Appendix.

## Results

### Base-case results

Based on the results of the model-based analysis (Table 2), the use of the Elecsys AMH Plus immunoassay was associated with 136 more true positive diagnoses of PCOS per year, compared with TVUS (6,721 vs. 6,584, respectively). However, the Elecsys AMH Plus immunoassay also identified more false positives than TVUS for AFC. The base-case results indicated that the Elecsys AMH Plus immunoassay could provide cost savings of £284,029 per year on the total cost of PCOS diagnosis, which equates to £22 per diagnosis per year. Furthermore, the component costs that could be avoided, per year, are £186,429 on consultations and £512,938 on TVUS. Although the cost of lifestyle interventions increases by £177,513 with the Elecsys AMH Plus immunoassay, £67,542 could be saved on T2D care and £98,567 could be saved on stroke care. In addition, use of the Elecsys AMH Plus immunoassay could reduce the number of cases of T2D and stroke per year.

**Table 2:** Number of diagnoses and costs per year from the NHS perspective in the UK, 2022.

	With TVUS (base-case)	With the AMH test^*^ (base-case)	Differences^†^
**PCOS diagnoses (per year), n**			
At primary care	12,987	12,987	0
At secondary care	0	0	0
**PCOS diagnoses per result type (per year), n**			
TP	6,584	6,721	136
FP	344	697	353
TN	5,478	5,125	−353
FN	581	444	−136
**New cases of T2D and stroke (per year), n**			
New cases of T2D	187	185	−2.0
New cases of stroke	205	203	−2.1
**Cost of PCOS (per year), £**			
Total	19,941,734	19,657,705	−284,029
Per diagnosis	1,536	1,514	−22
**Diagnosis of PCOS costs components (per year), £**			
Consultations/consultation time	761,180	574,751	−186,429
Laboratory costs	269,222	269,222	0
AFC with TVUS	512,938	0	−512,938
Elecsys AMH Plus immunoassay	0	403,934	403,934
**Other cost components (per year), £**			
Lifestyle interventions	2,512,189	2,689,702	177,513
T2D	6,459,543	6,392,000	−67,542
Stroke	9,426,662	9,328,095	−98,567

* The Elecsys AMH Plus immunoassay was used for the AMH test.

† All values for TVUS for AFC and Elecsys AMH Plus immunoassay have been rounded up to the nearest whole number for clarity. Consequently, the differences shown are based on the actual unrounded values, which may result in slight discrepancies when compared to the rounded values.

AFC, antral follicle count; AMH, anti-Müllerian hormone; FN, false negative; FP, false positive; NHS, National Health Service; PCOS, polycystic ovary syndrome; T2D, type 2 diabetes; TN, true negative; TP, true positive; TVUS, transvaginal ultrasound; UK, United Kingdom.

### Sensitivity and scenario analysis

**Fig 2:**
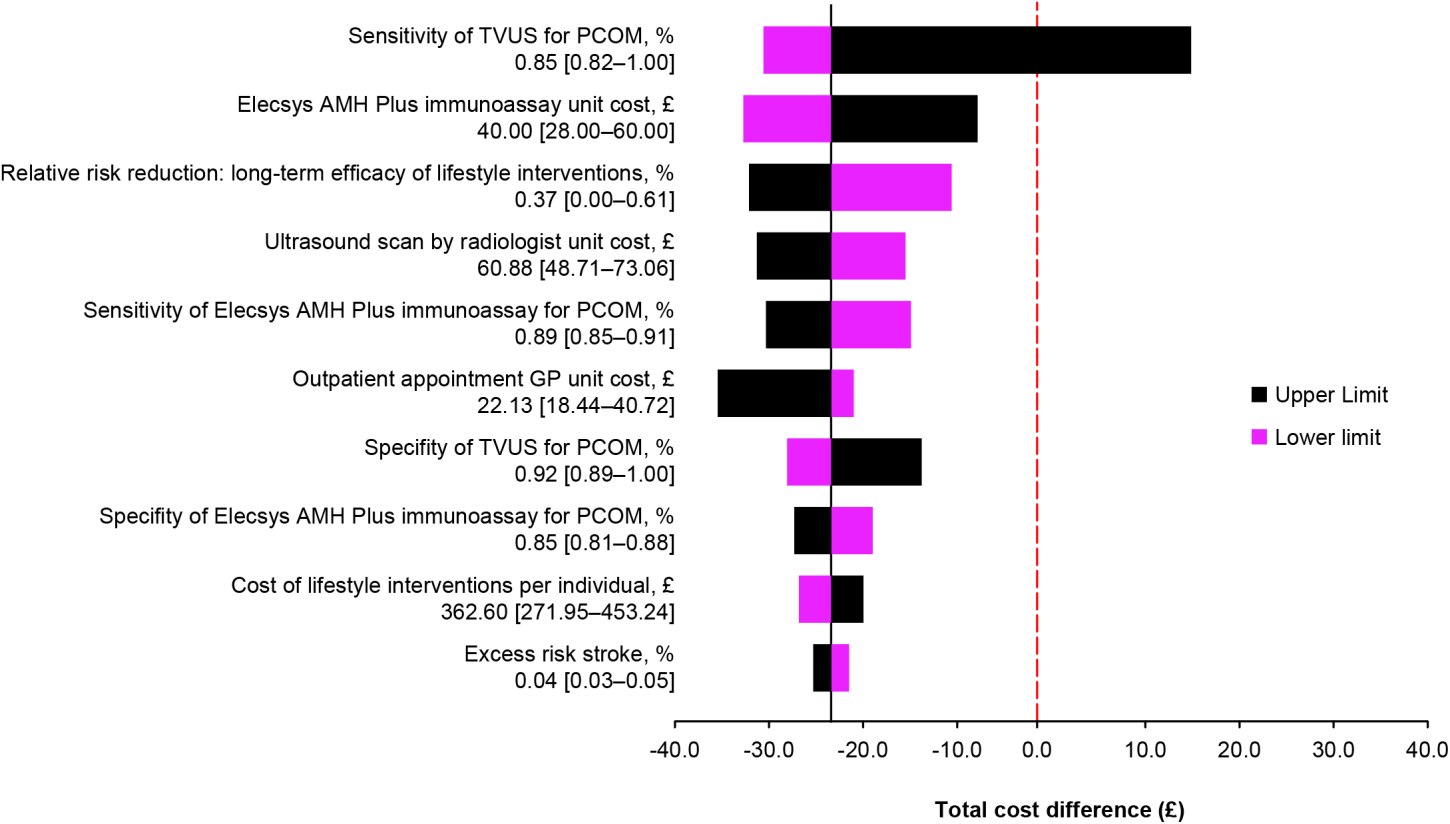
Deterministic sensitivity analysis illustrating the alteration from the base-case result if parameters are changed to the upper or lower limit using the NHS perspective in the UK, 2022. The vertical red dotted line denotes the point when the total cost difference between TVUS for AFC and the intervention is 0. The values in brackets are the variability ranges [min–max] from Table 1. AFC, antral follicle count; AMH, anti-Müllerian hormone; CI, confidence interval; GP, general practitioner; NHS, National Health Service; PCOM, polycystic ovarian morphology; TVUS, transvaginal ultrasound; UK, United Kingdom.

The sensitivity analysis (Fig 2) for the population suggested that the main deterministic variables of the model were: sensitivity of TVUS for the detection of PCOM, unit cost of the Elecsys AMH Plus immunoassay, relative risk reduction: long-term efficacy of lifestyle interventions, unit cost of TVUS, sensitivity of the Elecsys AMH Plus immunoassay for the detection of PCOM, unit cost of outpatient appointment with a GP, specificity of TVUS for the detection of PCOM, specificity of the Elecsys AMH Plus immunoassay for the detection of PCOM, cost of lifestyle interventions (per individual), and excess stroke risk. The greatest effector of the model was the sensitivity of TVUS for the detection of PCOM.

**Fig 3:**
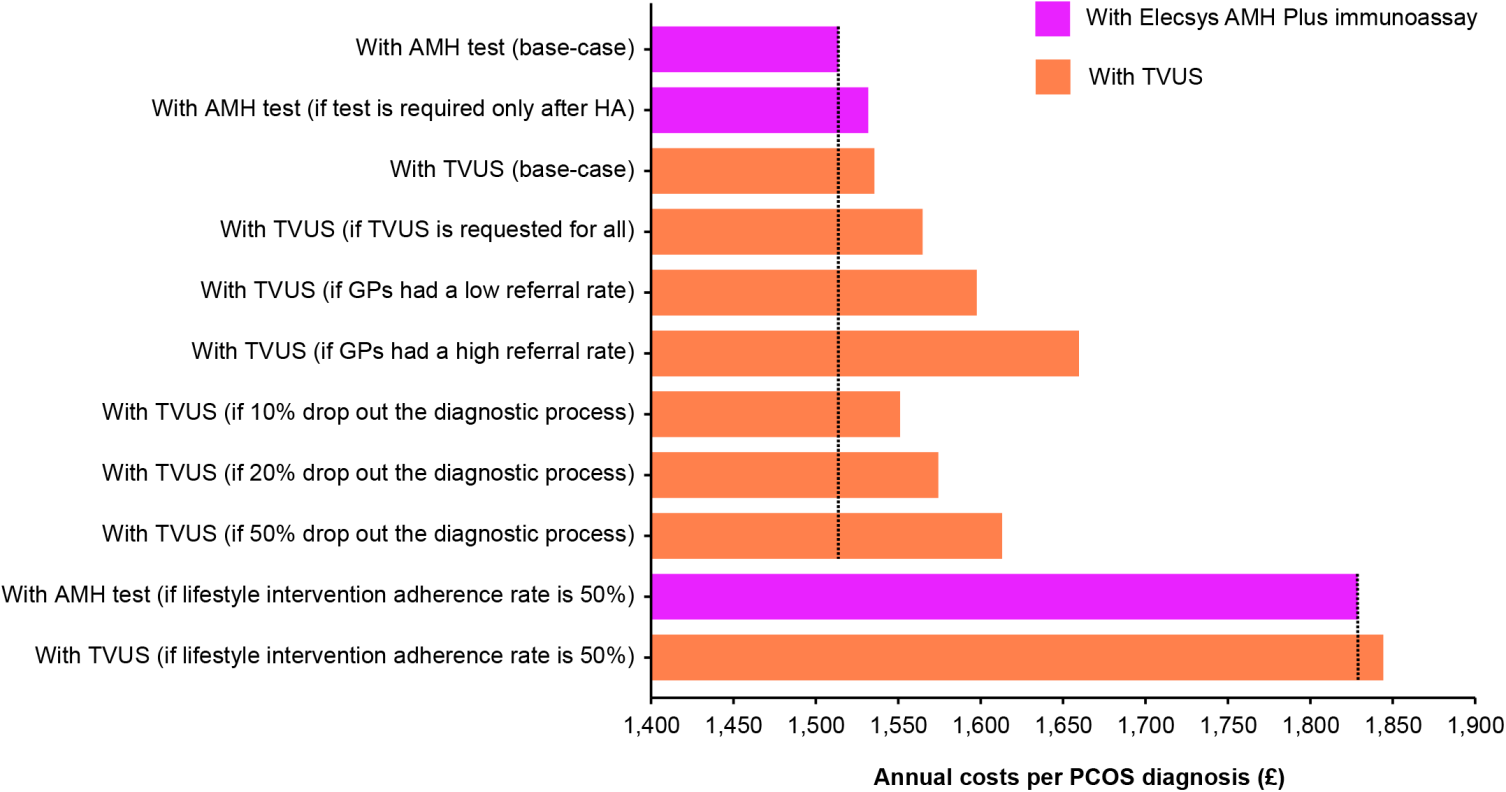
Annual cost per polycystic ovary syndrome diagnosis. **Base-case and scenario analyses for Elecsys AMH Plus immunoassay (illustrated in pink) and TVUS for AFC (illustrated in orange) from the NHS perspective in the UK, 2022.** The horizontal black dotted line facilitates the comparison between the Elecsys AMH Plus immunoassay base-case annual costs per PCOS diagnosis and the costs calculated by the other base-case and scenario analyses. AMH, anti-Müllerian hormone; GP, general practitioner; HA, hyperandrogenism; NHS, National Health Service; PCOS, polycystic ovary syndrome; TVUS, transvaginal ultrasound; UK, United Kingdom.

Based on the assumptions made by the model in the scenario analysis (Fig 3), the annual cost per PCOS diagnosis with the Elecsys AMH Plus immunoassay (base-case analysis) was £1,514. The results of the various scenario analyses using TVUS can be compared to the AMH test base-case analysis; the annual costs per PCOS diagnosis in all scenarios using TVUS were higher than those using the Elecsys AMH Plus immunoassay. For example, the annual cost of PCOS diagnosis using TVUS if requested for all patients was £1,565.

An additional scenario analysis was conducted to replace the calibration estimates with data from the HARMONIA study [31]. In general terms, after replacing the calibrated distribution of PCOS characteristics in patients with signs and symptoms of PCOS with sub-sample data from HARMONIA, the point estimate results show an increase in the savings from replacing TVUS with the Elecsys AMH Plus immunoassay. Further detail can be found in the Supporting Information, Supplementary Methods and S7–S9 Tables in S1 Appendix.

The probabilistic sensitivity analysis showed that a higher number of true positive PCOS diagnoses per year may be associated with overall cost savings (S2 Fig in S1 Appendix). A detailed breakdown of results for all scenario analyses is provided in the Supporting Information (S10–S17 Tables in S1 Appendix).

## Discussion

The aim of the current study was to explore the impact of implementing the Elecsys AMH Plus immunoassay to identify PCOM as part of PCOS assessment in women with signs and symptoms, from the perspective of the UK NHS. According to our estimates, implementing the Elecsys AMH Plus immunoassay in the UK would result in a greater number of PCOS diagnoses compared with TVUS for AFC and lower costs. In total, the cost savings of £284,029 per year could be made on the cost of PCOS diagnosis, in addition to savings on T2D care and stroke care.

Due to the established challenges associated with successfully diagnosing PCOS, including the limitations within the assessment of the current diagnostic criteria [17–21], the use of the Elecsys AMH Plus immunoassay as a substitute for determining PCOM is expected to be beneficial for the primary care setting in the UK. The addition of the Elecsys AMH Plus immunoassay to the roster of diagnostic tools offers numerous advantages, such as replacing the invasive TVUS, which requires an experienced sonographer [17], with a blood test that can be performed at the primary care level. The use of the Elecsys AMH Plus immunoassay may also increase accessibility for those who were previously hesitant to undergo TVUS due to cultural or religious reasons [20]. Provided that patients presenting with PCOS symptoms are referred to a gynecologist, the advantages of the Elecsys AMH Plus immunoassay could allow for timely management of PCOS, improvements in diagnosis for under-represented populations, and enable management of long-term, serious comorbidities that result from this condition.

In this model, the Elecsys AMH Plus immunoassay was less costly than TVUS (£22 cheaper per diagnosis), suggesting there is an economic advantage of its use in primary care settings, in addition to the clinical benefits. Furthermore, in the context of the UK NHS, a cheaper and faster diagnostic tool will likely enable a larger population to be tested compared with using TVUS. However, it should be noted that as the Elecsys AMH Plus immunoassay also identified more false positives than TVUS for AFC, a proportion of the cost savings might be offset.

In the UK NHS, women are currently referred to diagnostic centers for TVUS due to the long waiting periods in the primary care setting; delayed or missed PCOS diagnoses occur because of this [18,19]. In contrast, the Elecsys AMH Plus immunoassay can be performed at the same time as other blood tests in primary care, providing assessment sooner and thus avoiding these delays, as well as saving considerably on the costs associated with consultations and the costs of TVUS. The consideration of waiting times was not included in the current model due to the uncertainty around the impact this delay could have on health outcomes; further longitudinal studies are needed to understand the long-term impact of these delays on patients and the healthcare system.

Despite sub-fertility being one of the main symptoms of PCOS, women experiencing PCOS-related infertility were not included in this model due to the high uncertainty of the impact that the Elecsys AMH Plus immunoassay would have on the diagnostic pathway in this patient population. However, previous research has indicated that approximately half of patients with PCOS will seek infertility treatment, with an estimated annual cost of $533 million in 2004 in the United States, indicating that there is a notable cost associated with PCOS-related infertility [40]. The implementation of the Elecsys AMH Plus immunoassay therefore has the potential to result in further economic benefits with regard to this population, where an earlier diagnosis of PCOS could lead to earlier fertility treatment, assessment of comorbidities, and support with lifestyle interventions. Preventing the occurrence of obesity, or managing weight loss in those with the condition, has been found to improve ovulation and fertility [41–43], which may reduce the need for expensive fertility procedures. Further research should aim to quantify the economic benefits of the Elecsys AMH Plus immunoassay regarding women with PCOS-related infertility.

Our health economics model highlights the potential economic impact of implementing the Elecsys AMH Plus immunoassay in the UK NHS, based on recently published guidelines that allow the replacement of AFC determination by TVUS with AMH measurement for the assessment of PCOM in PCOS [23]. The model also explores the possibility that, after implementation, AMH levels could be assessed together with other blood work before the HA criterion is determined, enabling an earlier diagnosis. Further, potential savings from diagnosing PCOS, and therefore reducing the risk of secondary comorbidities, are considered. Finally, the model takes into account the performance of TVUS versus the Elecsys AMH Plus immunoassay, considering that AMH measurement is an automated assay, while TVUS is a highly operator-dependent procedure.

In this study, the Elecsys AMH Plus immunoassay was compared to the current TVUS for AFC for determining PCOM. The performance of TVUS used in our model was based on data published by the 2023 International Evidence-based Guidelines, which were derived from a meta-analysis of different publications and therefore different populations [44]. These data were mainly based on case-control studies, where cases consisted of all PCOS phenotypes and controls consisted of women without PCOS. Therefore, this population is not the same as the population that was used as part of the APHRODITE study, from which the performance of the Elecsys AMH Plus immunoassay was derived and validated. This discrepancy in the population between the studies used to support the performance of the Elecsys AMH Plus immunoassay versus TVUS is one of the limitations of this model. We believe, however, that as PCOS phenotype B (i.e., PCOS without PCOM) is quite limited, this discrepancy is unlikely to have a notable impact on the calculation of TVUS performance. In addition, it was assumed that no TVUS for AFC were performed when using the Elecsys AMH Plus immunoassay for PCOS assessment. This study examined AFC with TVUS; whilst it should be noted that some women who are tested with the Elecsys AMH Plus immunoassay may still need TVUS to explore other conditions/indications (either in the scenario with or without the Elecsys AMH Plus immunoassay), these instances were not included in the cost of assessing PCOS in this study.

Although our study did include a scenario with low adherence to lifestyle interventions, the estimated costs of lifestyle interventions used in our model were derived from a 12-week program conducted in Sheffield in the UK, as there was no clear guidance for the UK as part of the PCOS recommendations. However, it is likely that the cost of lifestyle interventions will vary depending on location (with rural/urban and North/South differences); therefore, further studies are needed to assess the costs and effectiveness of lifestyle interventions across the UK.

An assumption was made that if lifestyle interventions were adopted, the risk of developing secondary comorbidities, such as stroke and T2D, was reduced. The calculation of the reduction of the risk of stroke and T2D was based on a previous study of the prevention of T2D [45], rather than PCOS, and so it may not be directly transferrable to our study nor accurately reflect outcomes in PCOS [45]. Further studies are needed to establish the risk of developing secondary comorbidities following lifestyle interventions for PCOS, to confirm whether the savings in all scenarios using the Elecsys AMH Plus immunoassay for PCOS diagnosis in our study are accurate.

Another limitation is that the model in this study only focuses on the introduction of lifestyle changes as an intervention (treatment) for individuals diagnosed with PCOS, as this is usually the first recommendation provided [46]. Other treatments recommended based on PCOS-related symptoms were therefore not considered due to their recommendation being on a case-by-case basis. For instance, although the contraceptive pill is an intervention offered to individuals with PCOS [47], this was not included in the model as it can also be prescribed to individuals showing some of the signs or symptoms of PCOS but who do not suffer from the condition. Due to the varying lifestyle interventions used across studies of PCOS [46,48], as well as the general interventions suggested by the 2023 International Evidence-based Guidelines [44], our study focused on those that are frequently used in the primary healthcare setting in the UK. As such, the results of this study may not be reflective of interventions recommended in other countries.

Finally, as this study was funded by Roche Diagnostics International Ltd, the manufacturer of the Elecsys AMH Plus immunoassay, and includes Roche employees as authors, results should be interpreted in this context. However, we would emphasize that the study design, data collection, analysis, and interpretation were conducted according to scientific and ethical standards. All efforts were made to ensure scientific integrity, objectivity, and transparency throughout the process as per the International Committee of Medical Journal Editors guidelines. Interpretations of findings were driven by the collective input of the author group, not only Roche authors, in an independent manner.

In this health economics model, it was predicted that by utilizing the Elecsys AMH Plus immunoassay for diagnosing women with PCOS, the UK NHS would benefit by increasing the number of women diagnosed with PCOS, reducing the costs associated with PCOS diagnosis, and reducing the costs associated with common secondary comorbidities. With the known delays in the NHS for diagnosis of PCOS in many patients using the current TVUS for AFC, implementing the faster alternative Elecsys AMH immunoassay may not only provide cost benefits, but also reduce waiting times for diagnosis and treatment and lead to improved patient health outcomes.

## Supporting information

S1 Appendix Supplementary methods and results. (DOCX)
