# Supplementary material for: Economic impact of the Elecsys anti-Müllerian hormone Plus immunoassay for anti-Müllerian hormone testing as part of polycystic ovary syndrome assessment in the United Kingdom
Source: PLoS One. 2025 Jun 17;20(6):e0326162. doi: 10.1371/journal.pone.0326162 (PMC12173178; doi:10.1371/journal.pone.0326162)
Supplement: S1 Appendix — (DOCX) [file pone.0326162.s001.docx]

# Supplementary methods

## Calibration of parameters

In the absence of data, the incidence rate of signs and symptoms, and the distribution of polycystic ovary syndrome (PCOS) characteristics in women with signs and symptoms, were estimated through a calibration process. The calibration consisted of finding a combination for the distribution of PCOS characteristics and the incidence of signs and symptoms of PCOS so the model predictions matched a group of selected targets. The following targets were used for the calibration (S1 Table in Supplementary Appendix):

- PCOS characteristics, in patients with PCOS (unselected population), from Lizneva 2016 [1]
- PCOS characteristics, in patients with and without PCOS (with signs and symptoms of PCOS), from Gabrielli 2012 [2]
- Phenotype distribution, in patients with PCOS (unselected population), from Lizneva 2016 [1]
- PCOS incidence, from Liu 2021 [3]

**S1 Table. Defined targets for the calibration of parameters.**

|  | **Targets** | | **Source of targets** |
| --- | --- | --- | --- |
|  | **% or rates** | **n** |  |
| **PCOS characteristics, in patients with PCOS (unselected population)** | | | |
| Percentage with OA | 64.9% | 4,666 | Lizneva 2016 [1] |
| Percentage with HA | 80.4% | 5,778 | Lizneva 2016 [1] |
| Percentage with PCOM | 74.2% | 5,333 | Lizneva 2016 [1] |
| **PCOS characteristics, in patients with and without PCOS  (with signs and symptoms of PCOS)** | | | |
| Percentage with OA | 54.7% | 7,103 | Gabrielli 2012 [2] |
| Percentage with HA | 59.7% | 7,749 | Gabrielli 2012 [2] |
| Percentage with PCOM | — | — | — |
| **Phenotype distribution, in patients with PCOS (unselected population)** | | | |
| A: HA, OA, and PCOM | 19.6% | 1,407 | Lizneva 2016 [1] |
| B: HA, OA | 25.8% | 1,852 | Lizneva 2016 [1] |
| C: HA, PCOM | 35.1% | 2,518 | Lizneva 2016 [1] |
| D: OA, PCOM | 19.6% | 1,407 | Lizneva 2016 [1] |
| **PCOS incidence** | | | |
| Annual incident cases,  n per 100,000 | 78.6 | 7,185 | Liu 2021 [3] |

Values from Table 1 in the manuscript were normalized to sum 100%. HA, hyperandrogenism; OA, oligo-/anovulation; PCOM, polycystic ovarian morphology; PCOS, polycystic ovary syndrome.

The calibration process was conducted in accordance with the following steps:

1. Initially, the number of expected women with oligo-/anovulation (OA) that were exhibiting signs and symptoms was established, assuming a prevalence rate consistent with that observed in Gabrielli 2012 (54.7%) [2]
2. Subsequently, the number of women with hyperandrogenism (HA) in patients with signs and symptoms (for OA=negative and OA=positive) was estimated, with the objective of aligning the model predictions with the defined targets of PCOS characteristics in patients with and without PCOS from Gabrielli 2012 [2], as well as the phenotype distribution in patients with PCOS from Lizneva 2016 [1], which was derived from an unselected population
3. Step two was replicated for the distribution of polycystic ovarian morphology (PCOM) in patients with and without HA, and with and without OA
4. Finally, the incidence of signs and symptoms was calculated to match the expected incident cases of PCOS, according to the assumptions of steps one to three.

The final estimates for the calibration of parameters are summarized in S2 Table in Supplementary Appendix.

**S2 Table. Calibrated distribution of PCOS characteristics in the population with signs and symptoms.**

| **Distribution of characteristics in unselected population with signs and symptoms** | **% or  rates** | **n** |
| --- | --- | --- |
|  |  |  |
| Women aged 25–50 years | 100% | 9,145,746 |
| Incidence of signs and symptoms, n per 100,000 | 142 | 12,987 |
| OA_yes HA_yes  PCOM yes  PCOM no HA_no  PCOM yes  PCOM no | 54.7% 46.0% 44.0% 56.0% 54.0% 37.0% 63.0% | 7,103 3,268 1,438 1,830 3,836 1,419 2,417 |
| OA_no HA_yes  PCOM yes PCOM no | 45.3% 78.0% 54.0% 46.0% | 5,884 4,589 2,478 2,111 |

The numbers in red were calibrated and numbers in black (with the exception of the number of women aged 25–50 years old), were deduced after the calibration. HA, hyperandrogenism; OA, oligo-/anovulation; PCOM, polycystic ovarian morphology; PCOS, polycystic ovary syndrome.

The model predictions were compared against the selected targets after parameter calibration (S3 Table in Supplementary Appendix).

**S3 Table. Comparison between model predictions and defined targets after parameter calibration.**

|  | | | | | | | |
| --- | --- | --- | --- | --- | --- | --- | --- |
|  | **Model predictions** | | | **Targets** | | | **Source of targets** |
|  | **% or  rates** | **n** | | **% or  rates** | | **n** |  |
| **PCOS characteristics, in patients with PCOS (unselected population)** | | | | | | | |
| Percentage with OA | 65.4% | | 4,687 | 64.9% | 4,666 | | Lizneva 2016 [1] |
| Percentage with HA | 80.2% | | 5,746 | 80.4% | 5,778 | | Lizneva 2016 [1] |
| Percentage with PCOM | 74.5% | | 5,335 | 74.2% | 5,333 | | Lizneva 2016 [1] |
| **PCOS characteristics, in patients with and without PCOS (with signs and  symptoms of PCOS)** | | | | | | | |
| Percentage with OA | 54.7% | | 7,103 | 54.7% | 7,103 | | Gabrielli 2012 [2] |
| Percentage with HA | 60.5% | | 7,857 | 59.7% | 7,749 | | Gabrielli 2012 [2] |
| Percentage with PCOM | 41.1% | | 5,335 | — | — | | — |
| **Phenotype distribution, in patients with PCOS (unselected population)** | | | | | | | |
| A: HA, OA, and PCOM | 20.1% | | 1,438 | 19.6% | 1,407 | | Lizneva 2016 [1] |
| B: HA, OA | 25.5% | | 1,830 | 25.8% | 1,852 | | Lizneva 2016 [1] |
| C: HA, PCOM | 34.6% | | 2,478 | 35.1% | 2,518 | | Lizneva 2016 [1] |
| D: OA, PCOM | 19.8% | | 1,419 | 19.6% | 1,407 | | Lizneva 2016 [1] |
| **PCOS incidence** | | | | | | | |
| Annual incident cases,  n per 100,000 | 78.3 | | — | 78.6 | 7,185 | | Liu 2021 [3] |

HA, hyperandrogenism; OA, oligo-/anovulation; PCOM, polycystic ovarian morphology; PCOS, polycystic ovary syndrome.

## Meta-analysis: diagnostic performance of transvaginal ultrasound (TVUS) for PCOM

To estimate the diagnostic performance of TVUS for PCOM, a literature search was conducted with the objective of identifying studies reporting on diagnostic performance estimates of antral follicle count (AFC) measured by TVUS in order to identify cases of PCOM. The details of the search strategy are provided in S4 Table in Supplementary Appendix. The search yielded 34 articles, but all were excluded on the grounds of irrelevance. The majority of the studies focused on the performance of TVUS in diagnosing PCOS, rather than PCOM. In the absence of published data, our approach to estimate this parameter was to meta-analyze the articles used to discuss the diagnostic performance of TVUS for PCOM in the 2023 International Evidence-based Guidelines for the Assessment and Management of PCOS [4]. These guidelines faced the same problem, as articles were focused on PCOS, not PCOM. We believe, however, that as PCOS phenotype B (i.e. PCOS without PCOM) is quite limited, this discrepancy of PCOM versus PCOS is unlikely to have a notable impact on the results of this analysis. However, to compensate for the lack of data, we conservatively assumed in the 95% upper-level confidence intervals of the meta-analyzed estimations, a 100% diagnostic performance (i.e. 100% sensitivity and 100% specificity), instead of the meta-analyzed values of 87.5% and 95%, respectively (S6 Table in Supplementary Appendix). It is acknowledged that this approach may artificially overestimate the performance of TVUS for PCOM. However, given the lack of available data, this approach was deemed to be the most conservative option.

**S4 Table. Literature search strategy on diagnostic performance of TVUS for AFC.**

| Description: | **Literature search on performance of Antral Follicle Count (AFC) measured by Transvaginal Ultrasound (TVUS) to identify Polycystic Ovarian Morphology (PCOM)** | | |
| --- | --- | --- | --- |
| Focus: | **Performance of ultrasound measurement for AFC** | | |
| Methodology: | **Literature search on ProQuest Dialog in the following databases:**  Bibliographic content from Biosis, Embase Medline, Scisearch  Combination of Thesaurus terms (for Embase and Medline) and keyword search in Title, Abstract, Subject field | | |
|  | **Main concepts:**  Ultrasonography (thesaurus terms and several synonyms, like echography, sonography), in particular TVUS  Antral follicle count (thesaurus terms and synonyms, like follicle number)  PCOM (keyword search)  Sensitivity, area under the curve, performance (thesaurus terms and several synonyms) | | |
| Search strategy: | **Proquest Dialog** | | |
|  | # | Result(s) | Search query |
|  | S1 | 86516* | ((MESH.EXACT.EXPLODE("Ultrasonography, Prenatal") OR MESH.EXACT("Ultrasonography, Interventional")) OR (EMB.EXACT.EXPLODE("transvaginal echography"))) |
|  | S2 | 131219* | ((EMB.EXACT.EXPLODE("antral follicle count")) OR (MESH.EXACT.EXPLODE("Ovarian Follicle")) OR (ti,ab,su(((antral OR graafian OR tertiar* OR ovarian OR resting) N/1 (follicl* OR follicul*)) OR ((follicl* OR follicul*) N/1 (count* OR number*)) OR (ovar* N/1 reserv*) OR AFC))) |
|  | S3 | 60470* | ((ti,ab,su((ultrasound* OR ultrasonic* OR ultrasono* OR echograph* Or sonograph*) N/2 (*vagina* OR *follicl*) OR TVUS))) |
|  | S4 | 122197* | S3 OR S1 |
|  | S5 | 1075° | ((ti,ab,su((PCO N/1 morphol*) OR PCOM OR (polycystic* N/1 ovar* N/1 morphol*)))) |
|  | S6 | 22508576* | ((MESH.EXACT.EXPLODE("Sensitivity and Specificity") OR MESH.EXACT.EXPLODE("Area Under Curve")) OR (EMB.EXACT("error") OR EMB.EXACT.EXPLODE("sensitivity and specificity") OR EMB.EXACT.EXPLODE("area under the curve")) OR (ti,ab,su(sensitiv* OR specific* Or auc OR (area N/1 curve*) OR roc OR (analytic* N/1 valid*)))) |
|  | S7 | 23° | S6 AND S5 AND S4 AND S2 |
|  | S11 | 34118650* | ((MESH.EXACT.EXPLODE("Sensitivity and Specificity") OR MESH.EXACT.EXPLODE("Area Under Curve")) OR (EMB.EXACT("error") OR EMB.EXACT.EXPLODE("sensitivity and specificity") OR EMB.EXACT.EXPLODE("area under the curve")) OR (ti,ab,su(sensitiv* OR specific* Or auc OR (area N/1 curve*) OR roc OR (analytic* N/1 valid*) OR performan* OR precis* Or valid* OR accurat*))) |
|  | S12 | 27° | S11 AND S5 AND S4 AND S2 |
|  | S13 | 42357659* | ((MESH.EXACT.EXPLODE("Sensitivity and Specificity") OR MESH.EXACT.EXPLODE("Area Under Curve")) OR (EMB.EXACT("error") OR EMB.EXACT.EXPLODE("sensitivity and specificity") OR EMB.EXACT.EXPLODE("area under the curve")) OR (ti,ab,su(sensitiv* OR specific* Or auc OR (area N/1 curve*) OR roc OR (analytic* N/1 valid*) OR perform* OR precis* Or valid* OR accurat*))) |
|  | S14 | 34° | S13 AND S5 AND S4 AND S2 |
| * Duplicates are removed from the search but included in the result count. | | | |
| ° Duplicates are removed from the search and from the result count. | | | |

The meta-analysis was conducted using the software MetaDTA: Diagnostic Test Accuracy Meta-Analysis v2.1.3. S5 Table in Supplementary Appendix includes a comprehensive list of all of the studies included in the meta-analysis based on the studies that discussed diagnostic performance of TVUS for PCOM in the 2023 International Evidence-based Guidelines for the Assessment and Management of PCOS [4].

**S5 Table. Details on the studies included in the meta-analysis, including, year of publication, sensitivity, specificity, study sample size, women with PCOS, and controls.**

| **Study** | **Year** | **Sens** | **Spec** | **N** | **PCOS** | **Controls** |
| --- | --- | --- | --- | --- | --- | --- |
| Ahmad et al. 2019 (25–<30yo) [5] | 2019 | 0.73 | 0.7 | 353 | 125 | 228 |
| Ahmad et al. 2019 (30–<35yo) [5] | 2019 | 0.82 | 0.79 | 336 | 88 | 248 |
| Ahmad et al. 2019 (35–<40yo) [5] | 2019 | 0.75 | 0.83 | 312 | 32 | 280 |
| Allemand et al. 2006 [6] | 2006 | 0.7 | 1 | 39 | 10 | 29 |
| Carmina et al. 2016 [7] | 2016 | 0.93 | 0.85 | 160 | 113 | 47 |
| Chen et al. 2008 (MaxFN) [8] | 2008 | 0.852 | 0.926 | 585 | 432 | 153 |
| Christ et al. 2014 [9] | 2014 | 0.85 | 0.98 | 142 | 82 | 60 |
| Çiraci et al. 2015 [10] | 2015 | 0.854 | 0.895 | 96 | 48 | 48 |
| Dewailly et al. 2011 [11] | 2011 | 0.81 | 0.92 | 128 | 62 | 66 |
| Dewailly et al. 2014 [12] | 2014 | 0.832 | 0.925 | 716 | 95 | 621 |
| Diamanti-Kandarakis et al. 2011 [13] | 2011 | 0.85 | 0.98 | 97 | 50 | 47 |
| Jonard et al. 2005 [14] | 2005 | 0.79 | 0.97 | 211 | 154 | 57 |
| Sujata and Swoyam 2018 (2D) [15] | 2018 | 0.935 | 0.952 | 131 | 86 | 45 |
| Sujata and Swoyam 2018 (3D) [15] | 2018 | 0.88 | 0.977 | 131 | 86 | 45 |
| Köninger et al. 2014 (severe PCOS) [16] | 2014 | 0.881 | 0.896 | 107 | 59 | 48 |
| Köninger et al. 2014 (Mild PCOS) [16] | 2014 | 0.905 | 0.812 | 69 | 21 | 48 |
| Köşüş et al. 2011 [17] | 2011 | 0.95 | 1 | 310 | 210 | 100 |
| Lie Fong et al. 2017 (young) [18] | 2017 | 0.842 | 0.956 | — | 28 | 118 |
| Lie Fong et al. 2017 (old) [18] | 2017 | 0.795 | 0.935 | — | 51 | 100 |
| Lujan et al. 2013 [19] | 2013 | 0.85 | 0.94 | 168 | 98 | 70 |
| Villaroel et al. 2015 (adolescent) [20] | 2015 | 0.846 | 0.746 | 89 | 26 | 63 |
| Wongwananuruk et al. 2018 [21] | 2018 | 0.818 | 0.857 | 118 | 55 | 63 |

PCOS, polycystic ovary syndrome; sens, sensitivity; spec; specificity; yo, years old.

**S1 Fig. Random effects meta-analysis; sensitivity and specificity of TVUS for PCOS/PCOM.**


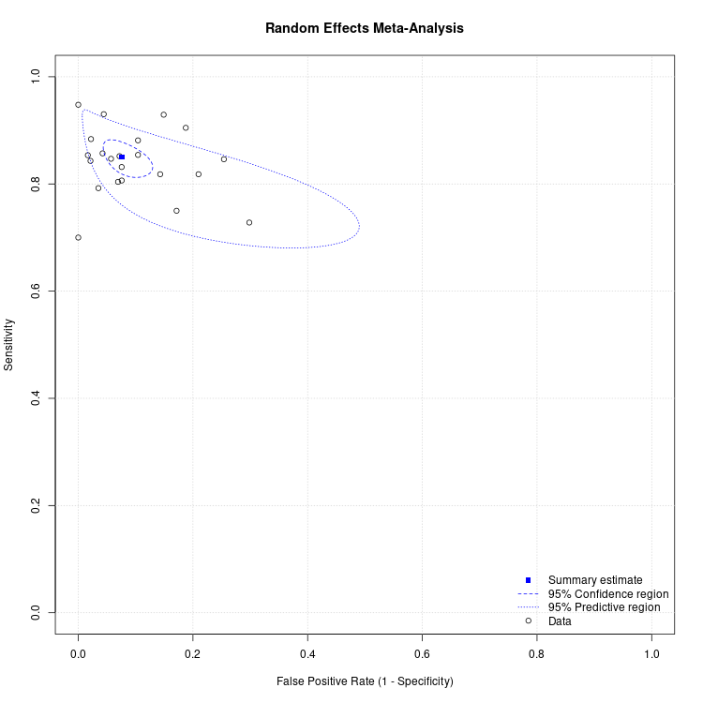


PCOM, polycystic ovarian morphology; PCOS, polycystic ovary syndrome; TVUS, transvaginal ultrasound.

**S6 Table. Meta-analyzed estimates of sensitivity and specificity of TVUS for PCOS/PCOM.**

| **Results** | **Estimate** | **2.5% CI** | **97.5% CI** |
| --- | --- | --- | --- |
| Sensitivity | 85.1% | 82.3% | 87.5% |
| Specificity | 92.4% | 88.7% | 95.0% |

CI, confidence interval; PCOM, polycystic ovarian morphology; PCOS, polycystic ovary syndrome; TVUS, transvaginal ultrasound.

## Scenario analysis: replacing the calibration estimates with data from the HARMONIA study

HARMONIA is a prospective study comprising an independent population of women born between July 1985 and December 1987 in Northern Finland [22]. During the development of this manuscript, data from the HARMONIA study became available, and therefore, in order to assess the potential impact of the calibration methods on the estimation of the distribution of PCOS characteristics in patients with signs and symptoms of PCOS, we replicated the entire analysis using a sub-sample of data from HARMONIA [22]. The sub-sample for our analysis consists of women with at least OA or HA, i.e. a proxy of signs and symptoms of PCOS. The distribution of characteristics is illustrated in S7 Table in Supplementary Appendix.

**S7 Table. PCOS characteristics in patients with signs and symptoms of PCOS from the HARMONIA study.**

| **OA** |  | **HA** |  | **PCOM** | **n (signs and symptoms)** |
| --- | --- | --- | --- | --- | --- |
| **OA_yes** | | | | | |
| OA_yes |  |  |  |  | 116 |
| OA_yes | and | HA_yes |  |  | 48 |
| OA_yes | and | HA_yes | and | PCOM yes | 37 |
| OA_yes | and | HA_yes | and | PCOM no | 9 |
| OA_yes | and | HA_yes | and | PCOM_unknown | 2 |
| OA_yes | and | HA_no |  |  | 68 |
| OA_yes | and | HA_no | and | PCOM yes | 33 |
| OA_yes | and | HA_no | and | PCOM no | 32 |
| OA_yes | and | HA_no | and | PCOM_unknown | 3 |
| **OA_no** | | | | | |
| OA_no |  |  |  |  | 178 |
| OA_no | and | HA_yes |  |  | 178 |
| OA_no | and | HA_yes | and | PCOM yes | 58 |
| OA_no | and | HA_yes | and | PCOM no | 112 |
| OA_no | and | HA_yes | and | PCOM_? | 8 |
| OA_no | and | HA_no |  |  | 843 |
| OA_no | and | HA_no | and | PCOM yes | 142 |
| OA_no | and | HA_no | and | PCOM no | 670 |
| OA_no | and | HA_no | and | PCOM_? | 31 |

Source: data on file, all women in whom it was possible to determine OA in the HARMONIA study. HA, hyperandrogenism; OA, oligo-/anovulation; PCOM, polycystic ovarian morphology; PCOS, polycystic ovary syndrome.

The resulting distribution of PCOS characteristics in patients with signs and symptoms of PCOS, defined as women with either OA or HA, from the HARMONIA study [22], are shown in S8 Table in Supplementary Appendix.

**S8 Table. The resulting distribution of PCOS characteristics in patients with signs and symptoms of PCOS from the HARMONIA study.**

| OA_yes HA_yes  PCOM yes  PCOM no HA_no  PCOM yes  PCOM no | 39.5% 41.4% 80.4% 19.6% 58.6% 50.8% 49.2% |
| --- | --- |
| OA_no HA_yes  PCOM yes  PCOM no | 60.5% 100.0% 34.1% 65.9% |

Source: data on file.
HA, hyperandrogenism; OA, oligo-/anovulation; PCOM, polycystic ovarian morphology; PCOS, polycystic ovary syndrome.

Finally, the results for the base-case and all scenario analyses were compiled in S9 Table in Supplementary Appendix. For simplicity, the table includes only the cost difference of PCOS per diagnosis, per year. In general terms, after replacing the calibrated distribution of PCOS characteristics in patients with signs and symptoms of PCOS with data from the HARMONIA study [22], the point estimate results show an increase in the savings from replacing TVUS by the anti-Müllerian hormone (AMH) test.

**S9 Table. Comparison of cost differences per PCOS, diagnosis, per year, with the calibration approach versus using data from HARMONIA.**

| **Results** | **With calibration** | **With HARMONIA data** |
| --- | --- | --- |
| **Base-case results, cost differences (per PCOS diagnosis,  per year), £** | -21.9 | -30.3 |
| **Scenario A:** With TVUS (if TVUS is requested for all) versus with the AMH test (base-case) | -51 | -43.8 |
| **Scenario B:** With TVUS (base-case) versus with the AMH test (if test is required only after HA) | -3.8 | -7.1 |
| **Scenario C:** With TVUS (if GPs had a low referral rate to specialists) versus with the AMH test (base-case) | -83.9 | -96.5 |
| **Scenario D:** With TVUS (if GPs had a high referral rate to specialists) versus with the AMH test (base-case) | -146 | -162.7 |
| **Scenario E:** With TVUS (considering a 10% drop out rate before TVUS) versus with the AMH test (base-case, no drop out) | -37.4 | -45.5 |
| **Scenario F:** With TVUS (considering a 25% drop out rate before TVUS) versus with the AMH test (base-case, no drop out) | -60.6 | -68.3 |
| **Scenario G:** With TVUS (considering a 50% drop out rate before TVUS) versus with the AMH test (base-case, no drop out) | -99.3 | -106.4 |
| **Scenario H:** Lower adherence rate for the lifestyle recommendations | -15.5 | -23.4 |

AMH, anti-Müllerian hormone; GP, General Practioner; PCOS, polycystic ovary syndrome; TVUS, transvaginal ultrasound.

# Supplementary results

**S2 Fig. 10,000 simulations of additional costs per diagnosis and additional true positive results, per year.**


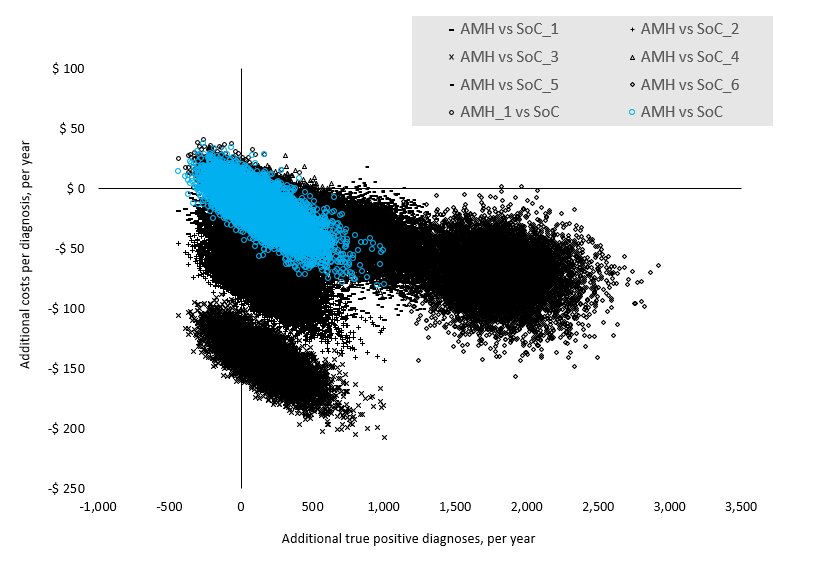


Base-case and scenario analyses. UK National Health Service perspective. United Kingdom, 2022. AMH, anti-Müllerian hormone; SoC, standard of care.

**S10 Table.** **Scenario A:** **with TVUS for AFC (if TVUS is requested for all PCOS suspicions) versus with the AMH test (base-case; after OA).**

|  | **With TVUS for AFC (if TVUS is requested for all)** | **With the AMH test* (base-case)** | **Differences^†^** |  |
| --- | --- | --- | --- | --- |
| **PCOS diagnoses (per year), n** | | | |  |
| At primary care | 12,987 | 12,987 | 0 |  |
| At secondary care | 0 | 0 | 0 |  |
| **PCOS diagnoses per result type (per year), n** | | | |  |
| TP | 6,584 | 6,721 | 136 |  |
| FP | 344 | 697 | 353 |  |
| TN | 5,478 | 5,125 | -353 |  |
| FN | 581 | 444 | -136 |  |
| **New cases of T2D and stroke (per year), n** | | | |  |
| New cases of T2D | 187 | 185 | -2 |  |
| New cases of stroke | 205 | 203 | -2 |  |
| **Cost of PCOS (per year), £** | | | |  |
| Total | 20,320,424 | 19,657,705 | -662,719 |  |
| Per diagnosis | 1,565 | 1,514 | -51 |  |
| **Diagnosis of PCOS costs components (per year), £** | | | |  |
| Consultations/consultation time | 862,126 | 574,751 | -287,375 |  |
| Laboratory costs | 269,222 | 269,222 | 0 |  |
| AFC with TVUS | 790,681 | 0 | -790,681 |  |
| Elecsys AMH Plus immunoassay | 0 | 403,935 | 403,935 |  |
| **Other cost components (per year), £** | | | |  |
| Lifestyle interventions | | 2,512,189 | 2,689,702 | 177,513 |
| T2D | | 6,459,543 | 6,392,000 | -67,542 |
| Stroke | | 9,426,662 | 9,328,095 | -98,567 |

*The Elecsys AMH Plus immunoassay was used for the AMH test. ^†^All values for TVUS for AFC and the Elecsys AMH Plus immunoassay have been rounded up to the nearest whole number for clarity. Consequently, the differences shown are based on the actual unrounded values, which may result in slight discrepancies when compared to the rounded values. AFC, antral follicle count; AMH, anti-Müllerian hormone; FN, false negative; FP, false positive; OA, oligo-/anovulation; PCOS, polycystic ovary syndrome; T2D, type 2 diabetes; TN, true negative; TP, true positive; TVUS, transvaginal ultrasound.

**S11 Table.** **Scenario B: with TVUS for AFC (base-case) versus with the AMH test (if test is required only after HA).**

|  | **With TVUS for AFC (base-case)** | **With the AMH test* (if test is required only after HA)** | **Differences^†^** |
| --- | --- | --- | --- |
| **PCOS diagnoses (per year), n** | | | |
| At primary care | 12,987 | 12,987 | 0 |
| At secondary care | 0 | 0 | 0 |
| **PCOS diagnoses per result type (per year), n** | | | |
| TP | 6,584 | 6,721 | 136 |
| FP | 344 | 697 | 353 |
| TN | 5,478 | 5,125 | -353 |
| FN | 581 | 444 | -136 |
| **New cases of T2D and stroke (per year), n** | | | |
| New cases of T2D | 187 | 185 | -2 |
| New cases of stroke | 205 | 203 | -2 |
| **Cost of PCOS (per year), £** | | | |
| Total | 19,941,734 | 19,892,744 | -48,990 |
| Per diagnosis | 1,536 | 1,532 | -4 |
| **Diagnosis of PCOS costs components (per year), £** | | | |
| Consultations/consultation time | 761,180 | 761,180 | 0 |
| Laboratory costs | 269,222 | 269,222 | 0 |
| AFC with TVUS | 512,938 | 0 | -512,938 |
| Elecsys AMH Plus immunoassay | 0 | 452,545 | 452,545 |
| **Other cost components (per year), £** | | | |
| Lifestyle interventions | 2,512,189 | 2,689,702 | 177,513 |
| T2D | 6,459,543 | 6,392,000 | -67,542 |
| Stroke | 9,426,662 | 9,328,095 | -98,567 |

*The Elecsys AMH Plus immunoassay was used for the AMH test. ^†^All values for TVUS for AFC and the Elecsys AMH Plus immunoassay have been rounded up to the nearest whole number for clarity. Consequently, the differences shown are based on the actual unrounded values, which may result in slight discrepancies when compared to the rounded values. AFC, antral follicle count; AMH, anti-Müllerian hormone; FN, false negative; FP, false positive; HA, hyperandrogenism; PCOS, polycystic ovary syndrome; T2D, type 2 diabetes; TN, true negative; TP, true positive; TVUS, transvaginal ultrasound.

**S12 Table. Scenario C: with TVUS for AFC (if GPs had a low referral rate to specialists) versus with the AMH test (base-case; after OA).**

|  | **With TVUS for AFC (if GPs had a low referral rate)** | **With the AMH test* (base-case)** | **Differences^†^** |
| --- | --- | --- | --- |
| **PCOS diagnoses (per year), n** | | | |
| At primary care | 11,039 | 12,987 | 1,948 |
| At secondary care | 1,948 | 0 | -1,948 |
| **PCOS diagnoses per result type (per year), n** | | | |
| TP | 6,584 | 6,721 | 136 |
| FP | 344 | 697 | 353 |
| TN | 5,478 | 5,125 | -353 |
| FN | 581 | 444 | -136 |
| **New cases of T2D and stroke (per year), n** | | | |
| New cases of T2D | 187 | 185 | -2 |
| New cases of stroke | 205 | 203 | -2 |
| **Cost of PCOS (per year), £** | | | |
| Total | 20,747,462 | 19,657,705 | -1,089,758 |
| Per diagnosis | 1,598 | 1,514 | -84 |
| **Diagnosis of PCOS costs components (per year), £** | | | |
| Consultations/consultation time | 1,567,249 | 574,751 | -992,498 |
| Laboratory costs | 268,882 | 269,222 | 341 |
| AFC with TVUS | 512,938 | 0 | -512,938 |
| Elecsys AMH Plus immunoassay | 0 | 403,935 | 403,935 |
| **Other cost components (per year), £** | | | |
| Lifestyle interventions | 2,512,189 | 2,689,702 | 177,513 |
| T2D | 6,459,543 | 6,392,000 | -67,542 |
| Stroke | 9,426,662 | 9,328,095 | -98,567 |

*The Elecsys AMH Plus immunoassay was used for the AMH test. ^†^All values for TVUS for AFC and the Elecsys AMH Plus immunoassay have been rounded up to the nearest whole number for clarity. Consequently, the differences shown are based on the actual unrounded values, which may result in slight discrepancies when compared to the rounded values. AFC, antral follicle count; AMH, anti-Müllerian hormone; FN, false negative; FP, false positive; GP, General Practitioner; OA, oligo-/anovulation; PCOS, polycystic ovary syndrome; T2D, type 2 diabetes; TN, true negative; TP, true positive; TVUS, transvaginal ultrasound.

**S13 Table.** **Scenario D: with TVUS for AFC (if GPs had a high referral rate to specialists) versus with the AMH test (base-case; after OA).**

|  | **With TVUS for AFC (if GPs had a high referral rate)** | **With the AMH test* (base-case)** | **Differences^†^** | |  |
| --- | --- | --- | --- | --- | --- |
| **PCOS diagnoses (per year), n** | | | | |  |
| At primary care | 9,091 | 12,987 | 3,896 | |  |
| At secondary care | 3,896 | 0 | -3,896 | |  |
| **PCOS diagnoses per result type (per year), n** | | | | |  |
| TP | 6,584 | 6,721 | 136 | |  |
| FP | 344 | 697 | 353 | |  |
| TN | 5,478 | 5,125 | -353 | |  |
| FN | 581 | 444 | -136 | |  |
| **New cases of T2D and stroke (per year), n** | | | | |  |
| New cases of T2D | 187 | 185 | -2 | |  |
| New cases of stroke | 205 | 203 | -2 | |  |
| **Cost of PCOS (per year), £** | | | | |  |
| Total | 21,553,191 | 19,657,705 | -1,895,486 | |  |
| Per diagnosis | 1,660 | 1,514 | -146 | |  |
| **Diagnosis of PCOS costs components (per year), £** | | | | |  |
| Consultations/consultation time | 2,373,318 | 574,751 | -1,798,567 | |  |
| Laboratory costs | 268,541 | 269,222 | 681 | |  |
| AFC with TVUS | 512,938 | 0 | -512,938 | |  |
| Elecsys AMH Plus immunoassay | 0 | 403,935 | 403,935 | |  |
| **Other cost components (per year), £** | | | | |  |
| Lifestyle interventions | | 2,512,189 | 2,689,702 | | 177,513 |
| T2D | | 6,459,543 | 6,392,000 | | -67,542 |
| Stroke | | 9,426,662 | 9,328,095 | | -98,567 |

*The Elecsys AMH Plus immunoassay was used for the AMH test. ^†^All values for TVUS for AFC and the Elecsys AMH Plus immunoassay have been rounded up to the nearest whole number for clarity. Consequently, the differences shown are based on the actual unrounded values, which may result in slight discrepancies when compared to the rounded values. AFC, antral follicle count; AMH, anti-Müllerian hormone; FN, false negative; FP, false positive; GP, General Practitioner; OA, oligo-/anovulation; PCOS, polycystic ovary syndrome; T2D, type 2 diabetes; TN, true negative; TP, true positive; TVUS, transvaginal ultrasound.

**S14 Table.** **Scenario E: with TVUS for AFC (considering a 10% drop out rate before TVUS) versus with the AMH test (base-case, no drop out; after OA).**

|  | **With TVUS for AFC (if 10% drop out the diagnostic process)** | **With the AMH test* (base-case)** | **Differences^†^** |
| --- | --- | --- | --- |
| **PCOS diagnoses (per year), n** | | | |
| At primary care | 12,987 | 12,987 | 0 |
| At secondary care | 0 | 0 | 0 |
| **PCOS diagnoses per result type (per year), n** | | | |
| TP | 6,253 | 6,721 | 468 |
| FP | 310 | 697 | 388 |
| TN | 5,512 | 5,125 | -388 |
| FN | 912 | 444 | -468 |
| **New cases of T2D and stroke (per year), n** | | | |
| New cases of T2D | 192 | 185 | -7 |
| New cases of stroke | 210 | 203 | -7 |
| **Cost of PCOS (per year), £** | | | |
| Total | 20,142,942 | 19,657,705 | -485,237 |
| Per diagnosis | 1,551 | 1,514 | -37 |
| **Diagnosis of PCOS costs components (per year), £** | | | |
| Consultations/consultation time | 742,537 | 574,751 | -167,786 |
| Laboratory costs | 269,222 | 269,222 | 0 |
| AFC with TVUS | 461,644 | 0 | -461,644 |
| Elecsys AMH Plus immunoassay | 0 | 403,935 | 403,935 |
| **Other cost components (per year), £** | | | |
| Lifestyle interventions | 2,379,450 | 2,689,702 | 310,252 |
| T2D | 6,623,767 | 6,392,000 | -231,767 |
| Stroke | 9,666,322 | 9,328,095 | -338,227 |

*The Elecsys AMH Plus immunoassay was used for the AMH test. ^†^All values for TVUS for AFC and the Elecsys AMH Plus immunoassay have been rounded up to the nearest whole number for clarity. Consequently, the differences shown are based on the actual unrounded values, which may result in slight discrepancies when compared to the rounded values. AFC, antral follicle count; AMH, anti-Müllerian hormone; FN, false negative; FP, false positive; OA, oligo-/anovulation; PCOS, polycystic ovary syndrome; T2D, type 2 diabetes; TN, true negative; TP, true positive; TVUS, transvaginal ultrasound.

**S15 Table.** **Scenario F: with TVUS for AFC (considering a 25% drop out rate before TVUS) versus with the AMH test (base-case, no drop out; after OA).**

|  | **With TVUS for AFC (if 25% drop out the diagnostic process)** | **With the AMH test*  (base-case)** | **Differences^†^** |
| --- | --- | --- | --- |
| **PCOS diagnoses (per year), n** | | | |
| At primary care | 12,987 | 12,987 | 0 |
| At secondary care | 0 | 0 | 0 |
| **PCOS diagnoses per result type (per year), n** | | | |
| TP | 5,755 | 6,721 | 966 |
| FP | 258 | 697 | 439 |
| TN | 5,564 | 5,125 | -439 |
| FN | 1,410 | 444 | -966 |
| **New cases of T2D and stroke (per year), n** | | | |
| New cases of T2D | 199 | 185 | -14 |
| New cases of stroke | 218 | 203 | -15 |
| **Cost of PCOS (per year), £** | | | |
| Total | 20,444,754 | 19,657,705 | -787,049 |
| Per diagnosis | 1,574 | 1,514 | -61 |
| **Diagnosis of PCOS costs components (per year), £** | | | |
| Consultations/consultation time | 714,573 | 574,751 | -139,822 |
| Laboratory costs | 269,222 | 269,222 | 0 |
| AFC with TVUS | 384,704 | 0 | -384,704 |
| Elecsys AMH Plus immunoassay | 0 | 403,935 | 403,935 |
| **Other cost components (per year), £** | | | |
| Lifestyle interventions | 2,180,341 | 2,689,702 | 509,361 |
| T2D | 6,870,104 | 6,392,000 | -478,104 |
| Stroke | 10,025,810 | 9,328,095 | -697,715 |

*The Elecsys AMH Plus immunoassay was used for the AMH test. ^†^All values for the TVUS for AFC and Elecsys AMH Plus immunoassay have been rounded up to the nearest whole number for clarity. Consequently, the differences shown are based on the actual unrounded values, which may result in slight discrepancies when compared to the rounded values. AFC, antral follicle count; AMH, anti-Müllerian hormone; FN, false negative; FP, false positive; OA, oligo-/anovulation; PCOS, polycystic ovary syndrome; T2D, type 2 diabetes; TN, true negative; TP, true positive; TVUS, transvaginal ultrasound.

**S16 Table.** **Scenario G: with TVUS for AFC (considering a 50% drop out rate before TVUS) versus with the AMH test (base-case, no drop out; after OA).**

|  | **With TVUS for AFC (if 50% drop out the diagnostic process)** | **With the AMH test*  (base-case)** | **Differences^†^** |
| --- | --- | --- | --- |
| **PCOS diagnoses (per year), n** | | | |
| At primary care | 12,987 | 12,987 | 0 |
| At secondary care | 0 | 0 | 0 |
| **PCOS diagnoses per result type (per year), n** | | | |
| TP | 4,926 | 6,721 | 1,795 |
| FP | 172 | 697 | 525 |
| TN | 5,650 | 5,125 | -525 |
| FN | 2,239 | 444 | -1,795 |
| **New cases of T2D and stroke (per year), n** | | | |
| New cases of T2D | 211 | 185 | -26 |
| New cases of stroke | 231 | 203 | -28 |
| **Cost of PCOS (per year), £** | | | |
| Total | 20,947,774 | 19,657,705 | -1,290,069 |
| Per diagnosis | 1,613 | 1,514 | -99 |
| **Diagnosis of PCOS costs components (per year), £** | | | |
| Consultations/consultation time | 667,965 | 574,751 | -93,214 |
| Laboratory costs | 269,222 | 269,222 | 0 |
| AFC with TVUS | 256,469 | 0 | -256,469 |
| Elecsys AMH Plus immunoassay | 0 | 403,935 | 403,935 |
| **Other cost components (per year), £** | | | |
| Lifestyle interventions | 1,848,493 | 2,689,702 | 841,209 |
| T2D | 7,280,666 | 6,392,000 | -888,665 |
| Stroke | 10,624,959 | 9,328,095 | -1,296,864 |

*The Elecsys AMH Plus immunoassay was used for the AMH test. ^†^All values for TVUS for AFC and the Elecsys AMH Plus immunoassay have been rounded up to the nearest whole number for clarity. Consequently, the differences shown are based on the actual unrounded values, which may result in slight discrepancies when compared to the rounded values. AFC, antral follicle count; AMH, anti-Müllerian hormone; FN, false negative; FP, false positive; OA, oligo-/anovulation; PCOS, polycystic ovary syndrome; T2D, type 2 diabetes; TN, true negative; TP, true positive; TVUS, transvaginal ultrasound.

**S17 Table.** **Scenario H: base-case AMH intervention (after OA) versus a scenario of TVUS for AFC with a 50% adherence rate to lifestyle recommendations.**

|  | **With TVUS for AFC (if lifestyle intervention adherence rate  is 50%)** | **With the AMH test* (if lifestyle intervention adherence rate is 50%)** | **Differences^†^** |
| --- | --- | --- | --- |
| **PCOS diagnoses (per year), n** | | | |
| At primary care | 12,987 | 12,987 | 0 |
| At secondary care | 0 | 0 | 0 |
| **PCOS diagnoses, per result type (per year), n** | | | |
| TP | 6,584 | 6,721 | 136 |
| FP | 344 | 697 | 353 |
| TN | 5,478 | 5,125 | -353 |
| FN | 581 | 444 | -136 |
| **New cases of T2D and stroke (per year), n** | | | |
| New T2D cases | 234 | 233 | -1.0 |
| New cases of stroke | 256 | 255 | -1.1 |
| **Cost of PCOS, (per year), £** | | | |
| Total | 23,950,641 | 23,749,667 | -200,974 |
| Per diagnosis | 1,844 | 1,829 | -16 |
| **Diagnosis of PCOS costs components (per year), £** | | | |
| Consultations/consultation time | 761,180 | 574,751 | -186,429 |
| Laboratory costs | 269,222 | 269,222 | 0 |
| AFC with TVUS | 512,938 | 0 | -512,938 |
| Elecsys AMH Plus immunoassay | 0 | 403,935 | 403,935 |
| **Other cost components (per year), £** | | | |
| Lifestyle interventions | 2,512,189 | 2,689,702 | 177,513 |
| T2D | 8,089,618 | 8,055,847 | -33,771 |
| Stroke | 11,805,494 | 11,756,211 | -49,284 |

*The Elecsys AMH Plus immunoassay was used for the AMH test. ^†^All values for TVUS for AFC and the Elecsys AMH Plus immunoassay have been rounded up to the nearest whole number for clarity. Consequently, the differences shown are based on the actual unrounded values, which may result in slight discrepancies when compared to the rounded values. AFC, antral follicle count; AMH, anti-Müllerian hormone; FN, false negative; FP, false positive; OA, oligo-/anovulation; PCOS, polycystic ovary syndrome; T2D, type 2 diabetes; TN, true negative; TP, true positive; TVUS, transvaginal ultrasound.

# References

1. Lizneva D, Kirubakaran R, Mykhalchenko K, Suturina L, Chernukha G, Diamond MP, et al. Phenotypes and body mass in women with polycystic ovary syndrome identified in referral versus unselected populations: systematic review and meta-analysis. Fertil Steril. 2016; 106: 1510-1520.e1512. <https://doi.org/10.1016/j.fertnstert.2016.07.1121>

2. Gabrielli L, Aquino EM. Polycystic ovary syndrome in Salvador, Brazil: a prevalence study in primary healthcare. Reprod Biol Endocrinol. 2012; 10: 96. <https://doi.org/10.1186/1477-7827-10-96>

3. Liu J, Wu Q, Hao Y, Jiao M, Wang X, Jiang S, et al. Measuring the global disease burden of polycystic ovary syndrome in 194 countries: Global Burden of Disease Study 2017. Hum Reprod. 2021; 36: 1108-1119. <https://doi.org/10.1093/humrep/deaa371>

4. Teede H, Tay CT, Laven J, Dokras A, Moran L, Piltonen T, et al. International evidence-based guideline for the assessment and management of polycystic ovary syndrome 2023. Melbourne, Australia, 2023.

5. Ahmad AK, Quinn M, Kao CN, Greenwood E, Cedars MI, Huddleston HG. Improved diagnostic performance for the diagnosis of polycystic ovary syndrome using age-stratified criteria. Fertil Steril. 2019; 111: 787-793.e782. <https://doi.org/10.1016/j.fertnstert.2018.11.044>

6. Allemand MC, Tummon IS, Phy JL, Foong SC, Dumesic DA, Session DR. Diagnosis of polycystic ovaries by three-dimensional transvaginal ultrasound. Fertil Steril. 2006; 85: 214-219. <https://doi.org/10.1016/j.fertnstert.2005.07.1279>

7. Carmina E, Campagna AM, Fruzzetti F, Lobo RA. AMH measurement versus ovarian ultrasound in the diagnosis of polycystic ovary syndrome in different phenotypes. Endocr Pract. 2016; 22: 287-293. <https://doi.org/10.4158/ep15903.Or>

8. Chen Y, Li L, Chen X, Zhang Q, Wang W, Li Y, et al. Ovarian volume and follicle number in the diagnosis of polycystic ovary syndrome in Chinese women. Ultrasound Obstet Gynecol. 2008; 32: 700-703. <https://doi.org/10.1002/uog.5393>

9. Christ JP, Willis AD, Brooks ED, Vanden Brink H, Jarrett BY, Pierson RA, et al. Follicle number, not assessments of the ovarian stroma, represents the best ultrasonographic marker of polycystic ovary syndrome. Fertil Steril. 2014; 101: 280-287.e281. <https://doi.org/10.1016/j.fertnstert.2013.10.001>

10. Çıracı S, Tan S, Özcan A, Aslan A, Keskin HL, Ateş Ö F, et al. Contribution of real-time elastography in diagnosis of polycystic ovary syndrome. Diagn Interv Radiol. 2015; 21: 118-122. <https://doi.org/10.5152/dir.2014.14094>

11. Dewailly D, Gronier H, Poncelet E, Robin G, Leroy M, Pigny P, et al. Diagnosis of polycystic ovary syndrome (PCOS): revisiting the threshold values of follicle count on ultrasound and of the serum AMH level for the definition of polycystic ovaries. Hum Reprod. 2011; 26: 3123-3129. <https://doi.org/10.1093/humrep/der297>

12. Dewailly D, Alebić M, Duhamel A, Stojanović N. Using cluster analysis to identify a homogeneous subpopulation of women with polycystic ovarian morphology in a population of non-hyperandrogenic women with regular menstrual cycles. Hum Reprod. 2014; 29: 2536-2543. <https://doi.org/10.1093/humrep/deu242>

13. Diamanti-Kandarakis E, Livadas S, Katsikis I, Piperi C, Mantziou A, Papavassiliou AG, et al. Serum concentrations of carboxylated osteocalcin are increased and associated with several components of the polycystic ovarian syndrome. J Bone Miner Metab. 2011; 29: 201-206. <https://doi.org/10.1007/s00774-010-0211-2>

14. Jonard S, Robert Y, Dewailly D. Revisiting the ovarian volume as a diagnostic criterion for polycystic ovaries. Hum Reprod. 2005; 20: 2893-2898. <https://doi.org/10.1093/humrep/dei159>

15. Sujata K, Swoyam S. 2D and 3D trans-vaginal sonography to determine cut-offs for ovarian volume and follicle number per ovary for diagnosis of polycystic ovary syndrome in Indian women. J Reprod Infertil. 2018; 19: 146-151.

16. Köninger A, Koch L, Edimiris P, Enekwe A, Nagarajah J, Kasimir-Bauer S, et al. Anti-Mullerian hormone: an indicator for the severity of polycystic ovarian syndrome. Arch Gynecol Obstet. 2014; 290: 1023-1030. <https://doi.org/10.1007/s00404-014-3317-2>

17. Köşüş N, Köşüş A, Turhan NO. Relationship of ovarian volume with mean platelet volume and lipid profile in patients with polycystic ovary syndrome. Exp Ther Med. 2011; 2: 1144. <https://doi.org/10.3892/etm.2011.327>

18. Lie Fong S, Laven JSE, Duhamel A, Dewailly D. Polycystic ovarian morphology and the diagnosis of polycystic ovary syndrome: redefining threshold levels for follicle count and serum anti-Müllerian hormone using cluster analysis. Hum Reprod. 2017; 32: 1723-1731. <https://doi.org/10.1093/humrep/dex226>

19. Lujan ME, Jarrett BY, Brooks ED, Reines JK, Peppin AK, Muhn N, et al. Updated ultrasound criteria for polycystic ovary syndrome: reliable thresholds for elevated follicle population and ovarian volume. Hum Reprod. 2013; 28: 1361-1368. <https://doi.org/10.1093/humrep/det062>

20. Villarroel C, López P, Merino PM, Iñiguez G, Sir-Petermann T, Codner E. Hirsutism and oligomenorrhea are appropriate screening criteria for polycystic ovary syndrome in adolescents. Gynecol Endocrinol. 2015; 31: 625-629. <https://doi.org/10.3109/09513590.2015.1025380>

21. Wongwananuruk T, Panichyawat N, Indhavivadhana S, Rattanachaiyanont M, Angsuwathana S, Techatraisak K, et al. Accuracy of anti-Müllerian hormone and total follicles count to diagnose polycystic ovary syndrome in reproductive women. Taiwan J Obstet Gynecol. 2018; 57: 499-506. <https://doi.org/10.1016/j.tjog.2018.06.004>

22. Piltonen TT, Allegranza D, Hund M, Buck K, Sillman J, Arffman RK. Validation of an anti-müllerian hormone cutoff for polycystic ovarian morphology in the diagnosis of polycystic ovary syndrome in the HARMONIA study: protocol for a prospective, noninterventional study. JMIR Res Protoc. 2024; 13: e48854. <https://doi.org/10.2196/48854>
